# Antibacterial and anticancer PDMS surface for mammalian cell growth using the Chinese herb extract paeonol(4-methoxy-2-hydroxyacetophenone)

**DOI:** 10.1038/srep38973

**Published:** 2016-12-12

**Authors:** Jiajia Jiao, Lili Sun, Zaiyu Guo, Sen Hou, Robert Holyst, Yun Lu, Xizeng Feng

**Affiliations:** 1 State Key Laboratory of Medicinal Chemical Biology, The Key Laboratory of Bioactive Materials, Ministry of Education, College of Life Science, Nankai University, Tianjin, 300071, China; 2 Institute of Physical Chemistry Polish Academy of Sciences, Kasprzaka 44/52, 01-224 Warsaw, Poland; 3 TEDA Hospital, No. 65 Third Avenue, Economic-Technological Development Area, Tianjin, 300457, China

## Abstract

Polydimethylsiloxane (PDMS) is widely used as a cell culture platform to produce micro- and nano-technology based microdevices. However, the native PDMS surface is not suitable for cell adhesion and is always subject to bacterial pollution and cancer cell invasion. Coating the PDMS surface with antibacterial or anticancer materials often causes considerable harm to the non-cancer mammalian cells on it. We have developed a method to fabricate a biocompatible PDMS surface which not only promotes non-cancer mammalian cell growth but also has antibacterial and anticancer activities, by coating the PDMS surface with a Chinese herb extract, paeonol. Coating changes the wettability and the elemental composition of the PDMS surface. Molecular dynamic simulation indicates that the absorption of paeonol onto the PDMS surface is an energy favourable process. The paeonol-coated PDMS surface exhibits good antibacterial activity against both Gram-positive and Gram-negative bacteria. Moreover considerable antibacterial activity is maintained after the coated surface is rinsed or incubated in water. The coated PDMS surface inhibits bacterial growth on the contact surface and promotes non-cancer mammalian cell growth with low cell toxicity; meanwhile the growth of cancer cells is significantly inhibited. Our study will potentially guide PDMS surface modification approaches to produce biomedical devices.

Polydimethylsiloxane (PDMS) is mechanically stable, non-toxic and elastic polymeric material^1^. It is widely used in biomedical devices including oxygenators, contact lenses, blood pumps, etc.^2^. However, the hydrophobic nature of PDMS inhibits cell adhesion and growth on its surface^3^. Improving the biocompatibility of the PDMS surface for mammalian cell growth is a challenging task for its application as a cell culture platform in biomedical devices^4^. Meanwhile uncontrolled bacterial adhesion and growth and cancer cell invasion often cause considerable harm to the non-cancer mammalian cells growing on the PDMS surface. Various techniques have been developed for the sterilization of PDMS surface. PDMS surfaces coated with antibacterial materials, such as titania and chlorogenic acid, are reported to have good antibacterial activity^5^,^6^. However, these materials are usually either poorly effective against cancer cells or highly toxic to non-cancer mammalian cells. A method that can promote mammalian cell growth on PDMS surface with good antibacterial and anticancer activities is required.

The traditional Chinese herb extract, paeonol (4-methoxy-2-hydroxyacetophenone), is expected to solve the problem. Paeonol is the active component, extracted from the Chinese herb *Paeonia moutan*^7^. *Paeonia moutan* has various pharmacological effects such as antioxidant, anti-inflammatory and immuno-regulatory activities^8^,^9^. Paeonol in solution is reported to exhibit considerable antibacterial and anticancer activities. The antibacterial effect results from paeonol’s ability to inhibit the α-glucosidase activity of bacteria and then induce cell death^10^,^11^. Paeonol inhibits the proliferation of tumor cell lines, such as T6-17, HeLa and HT-29^12^. It is also reported to be nontoxic to normal mammalian cells and sometimes even to benefit their growth. For example, Juliana *et al*. reported that paeonol is not only non-cytotoxic for endothelial cells, but also protects them from premature senescence by modulating the Sirtuin 1 pathway^13^. Ji Biao *et al*. reported that paeonol protects hippocampal neurons which are subjected to oxygen–glucose deprivation induced injury^14^. The effective antibacterial and anticancer properties and its low toxicity to normal mammalian cells make paeonol a potential coating material for the PDMS surface.

In this study, we developed a method of coating the PDMS surface with the traditional Chinese herbal extract paeonol to provide the surface with multiple functions: antibacterial, anticancer and promoting mammalian cell growth. *E. coli* DH5α, *P. aeruginosa* PAO1, HeLa cells and NIH3T3 cells were used as Gram-negative bacteria, Gram-positive bacteria, cancer cells and non-cancer mammalian cells respectively to examine the antibacterial and anticancer effects of the paeonol-coated PDMS surface. The coated PDMS surface was evaluated by water contact angle (WCA) and X-ray photoelectron spectroscopy (XPS) measurement. The approach of paeonol onto the PDMS surface was simulated by molecular dynamics (MD) simulation. The stability of the coated PDMS surfaces was studied by their anti-rinsing and anti-incubation properties. We studied the influence of paeonol concentration on the sterilizing effect of the coated PDMS surface. We studied the sterilizing effect of the coated PDMS surface on other surfaces by contact. The promoted growth of non-cancer mammalian cells on the coated PDMS surface and its anticancer effect were also studied.

## Results and Discussion

### Inhibition of bacterial growth by paeonol in solution

**Figure 1:**
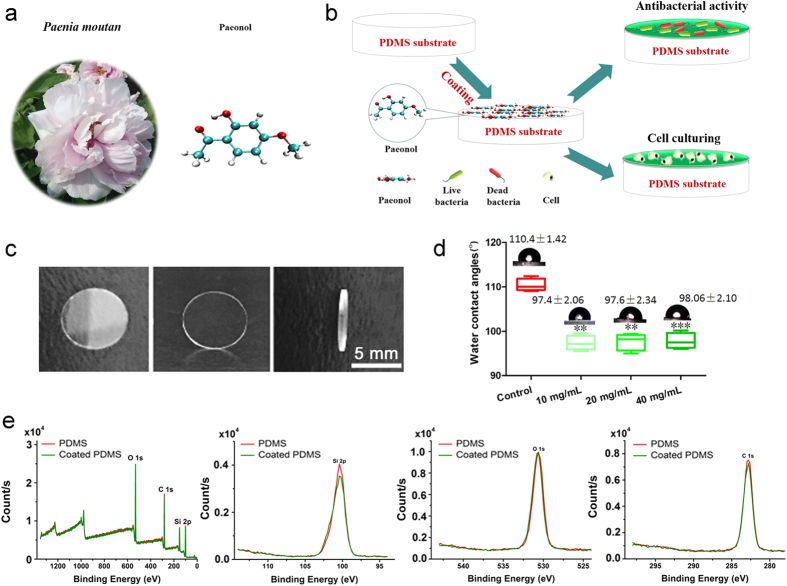
(**a**) The chemical structure of paeonol extracted from the Chinese herb *Paeonia moutan.* The O atom is depicted in red, the C atom is in green and the H atom is in white. (**b**) The surface modification procedure on the PDMS surface with paeonol. (**c**) The size of the PDMS disks used in the study. (**d**) The wettability of the paeonol-coated PDMS surface with a series of paeonol concentrations. (**e**) The XPS measurement of the paeonol-coated PDMS surface.

It has been documented that the Chinese traditional herb *Paeonia moutan* is effective against inflammation^8^. Its extract, paeonol (Fig. 1a), has been reported to have an antibacterial effect in solution^10^. We coated the PDMS surface with paeonol to promote mammalian cell growth and studied its antibacterial and anticancer activities as well as its toxicity to non-cancer mammalian cells (Fig. 1b). The PDMS was made into disks of 7 mm in diameter (Fig. 1c). Coating the PDMS surface with paeonol increased the surface wettability which is shown as a decrease of WCA from ca. 110° to 97° (Fig. 1d). The WCAs of the resulting PDMS surfaces were similar to each other when they were coated with a wide range of concentrations (from 10 mg/ml to 40 mg/ml) of paeonol solutions. The result indicates that the coated PDMS surfaces with similar properties can be produced by using paeonol solutions of different concentrations (see also the similar antibacterial effects of these surfaces). Thus the method is flexible in choosing the concentration of paeonol solutions. The relative elemental compositions of the PDMS surface changed after coating with paeonol. We observed a slight increase in O1s and a decrease in C1s and Si2p (Fig. 1e and Table S1). The relative change of elemental composition is supposedly caused by and can also be deemed as a sign of the coating of the PDMS surface with paeonol. The WCA and XPS results were confirmed by Fourier transform infrared spectroscopy (FTIR) measurement (Fig. S1).

### Molecular modelling and molecular dynamics simulations

The attachment of paeonol to the PDMS surface is the key step for downstream surface functionalization. We experimentally confirmed the attachment of paeonol onto the PDMS surface by WCA, XPS and FTIR measurements (Fig. 1d,e, S1 and Table S1); yet many details in the process are still unknown. Why do paeonol molecules tend to attach to the PDMS surface? How are paeonol molecules positioned on the PDMS surface? What kind of energy contributes to the attachment process? These questions need to be studied on a single-molecular level, which is difficult to realize by experiments. In this study we used molecular dynamics simulation to study the details of the attachment process.

**Figure 2:**
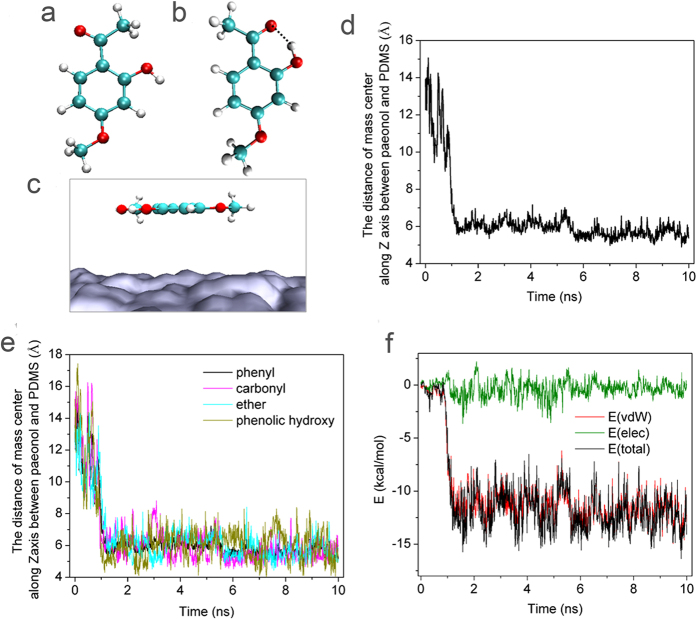
(**a**,**b**) The two conformers of paeonol. (**c**) The initial arrangement of paeonol on the PDMS surface in the molecular dynamics simulation. The O atom is depicted in red, the C atom is in green and the H atom is in white. The PDMS is in grey. (**d**) The time evolution of the distance between the mass center of the paeonol and the mass center of the PDMS substrate along the direction perpendicular to the PDMS substrate. (**e**) The time evolution of the distance between the mass centers of different chemical groups in paeonol and the mass center of the PDMS substrate along the direction perpendicular to the PDMS substrate. (**f**) The energy contribution of the van der Waals interaction and electrostatic interaction in the approach process.

The paeonol molecule has two major conformers. We built and optimized the favourable conformation (Fig. 2a–c). In conformer (a), the three O atoms are the furthest from each other, while no hydrogen bond is formed. In conformer (b), a hydrogen bond is formed between the hydroxyl group and the carbonyl group to stabilize the conformer. In the hydrogen bond, the distance of O∙∙∙O between the hydroxyl group and the carbonyl group is 2.558 Å and the O–H∙∙∙O angle is 149.2° (Fig. 2b). The energy of conformer (b) is 9.412 kcal/mol lower than conformer (a). Therefore, we chose conformer (b) as the initial conformation in the molecular dynamics simulation.

We positioned paeonol conformer (b) above the PDMS surface with an initial distance of 14 Å between the mass centre of the two. The initial arrangement of paeonol on the surface of the PDMS substrate is shown in Fig. 2c, where all C atoms and O atoms are coplanar (X-Y plane) and parallel with the surface of the PDMS substrate. The initial position of paeonol was 14 Å away from the PDMS along the direction perpendicular to the PDMS substrate (in the direction of the Z axis). During the simulation process, the paeonol automatically approached the PDMS surface (Fig. 2d). In the first 1 ns, the paeonol approached the PDMS surface rapidly. From 1 ns to 5.5 ns, the distance between mass centers along the Z axis remained stable at ca. 6 Å. From 5.5 ns to 10 ns, the distance between the mass centers along the Z axis was 5.61 Å. Since the distance from the mass center of PDMS to its surface was 5.5 Å, we expected that the paeonol had already closely contacted the PDMS surface after 5.5 ns.

We probed the orientation of the paeonol molecule on the PDMS surface by calculating the distance between the mass centers of different chemical groups in the paeonol and the mass center of the PDMS along the Z axis. The paeonol molecule is composed of four chemical groups: phenyl (-C_6_H_3_), phenolic hydroxyl (-OH), carbonyl (CH_3_CO-) and ether (CH_3_O-). During the adsorption process, these groups approached the PDMS surface competitively (Fig. 2e). After 5.5 ns, the approach process was stabilized. The phenolic hydroxyl group was the furthest from the surface of the PDMS with the largest standard deviation (phenyl 5.61 ± 0.32 Å, carbonyl 5.43 ± 0.47 Å, ether 5.67 ± 0.58 Å, phenolic hydroxyl 6.00 ± 0.95 Å). The phenolic hydroxyl group is hydrophilic, and the PDMS surface is hydrophobic. The result demonstrates that the hydrophilic phenolic hydroxyl group is not as favourable as other hydrophobic groups when positioning on the hydrophobic PDMS surface.

We probed the possibility of hydrogen bond formation during the adsorption process. We set the criterion for hydrogen bond formation as the O∙∙∙O distance between the donor and the acceptor less than 3.5 Å and the O–H∙∙∙O angle in the range of 150° ~ 180°. It was found that no hydrogen bond should form between the paeonol molecule and the PDMS surface. Therefore the hydrogen bond does not play a role in the adsorption process.

We calculated the contribution of van der Waals interaction and electrostatic interaction in the attachment process. As shown in Fig. 2 f, the total interaction energy is mainly contributed by van der Waals interaction. The electrostatic interaction contributes slightly to the adsorption process.

### Antibacterial property

**Figure 3:**
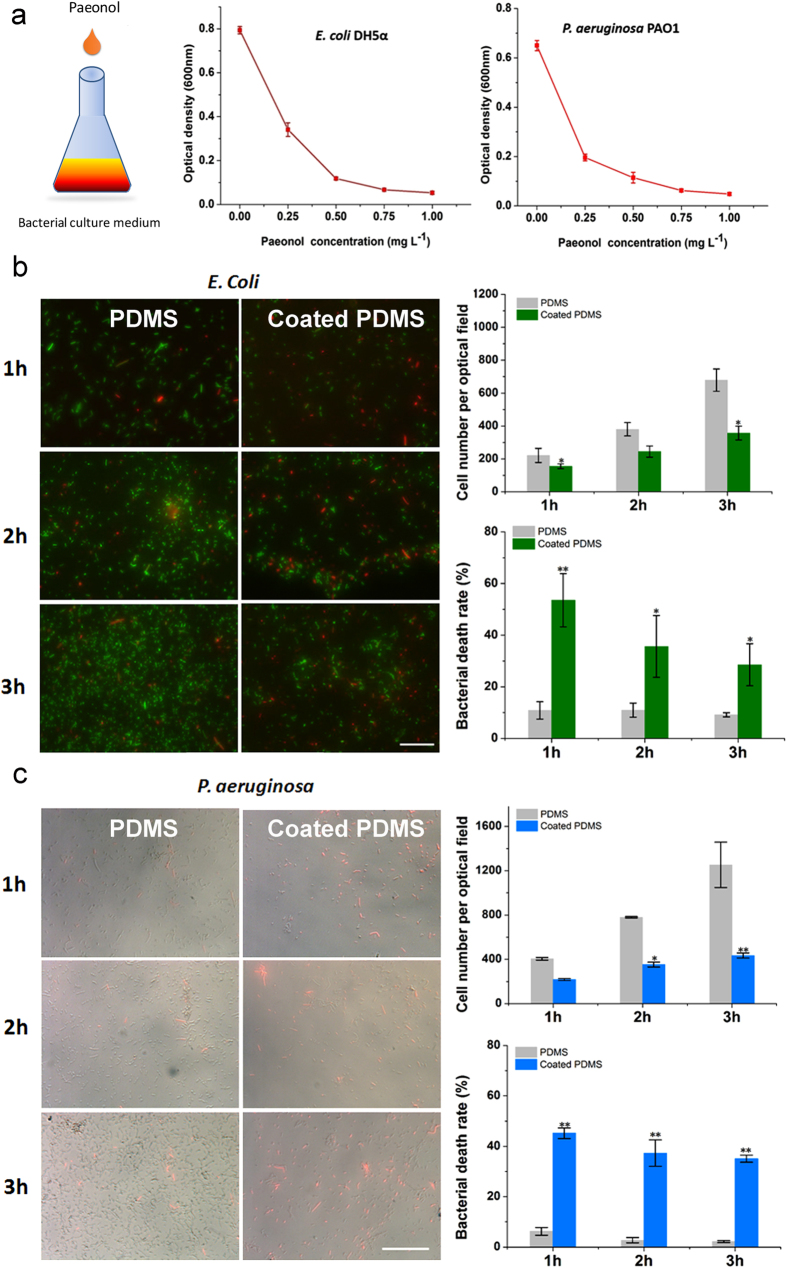
(**a**) Antibacterial effect of paeonol tested by *E. coli* DH5α and *P. aeruginosa* PAO1 in solutions. (**b**) Antibacterial effect of the paeonol-coated PDMS surface tested by *E. coli* DH5α. *E. coli* DH5α were genetically engineered to express green fluorescent proteins. (**c**) Antibacterial effect of the paeonol-coated PDMS surface tested by *P. aeruginosa* PAO1. The dead bacteria were stained using PI to be red. We show the fluorescent microscopic images on the left part in (**b**,**c**). The bacterial death rate and cell number (on the right) were calculated with at least 3 experimental repetitions. The difference between the paeonol-coated and uncoated PDMS samples was tested by Student’s *t*-test (*0.01 < p < 0.05, **0.001 < p < 0.01). The length of scale bar is 100 μm.

Since *E. coli* DH5α has a high resistance to antibiotics^6^, we used *E. coli* DH5α as the Gram-negative bacteria to check the antibacterial effect of paeonol. We also used *P. aeruginosa* PAO1 as the Gram-positive bacteria in the study. Bacterial growth was reduced by 94% for *E. coli* and by 92% for *P. aeruginosa* in the presence of 1 mg/ml paeonol in solution (Fig. 3a).

Although paeonol solution is an ideal reagent to kill bacteria, whether the paeonol-coated PDMS surface can still be active is unknown. In this study, the antibacterial activity of the paeonol-coated PDMS surface was quantitatively evaluated by bacterial number and bacterial death rate. Compared with the uncoated PDMS surface, the number of *E. coli* on the paeonol-coated PDMS surface decreased by ca. 50% and the bacterial death rate increased by ca. 3 times after 3 h (Fig. 3b). The number of *P. aeruginosa* on the paeonol-coated PDMS surface decreased by ca. 60% and the bacterial death rate increased by ca. 15 times after 3 h (Fig. 3c). The result indicates that the paeonol-coated PDMS surface is effective against both Gram-positive and Gram-negative bacteria.

Usually bacteria can mutate and adapt to a harsh environment in a few hours^15^. As a result, the bacteria become more resistant to paeonol and cell death rate should decrease. Although the cell death rate in our study did decrease, an appreciable cell death rate (~40%) was still observed after 3 h. This indicates that paeonol is an ideal material for PDMS modification with a strong antibacterial effect.

### Stability of the paeonol-coated PDMS surface

**Figure 4:**
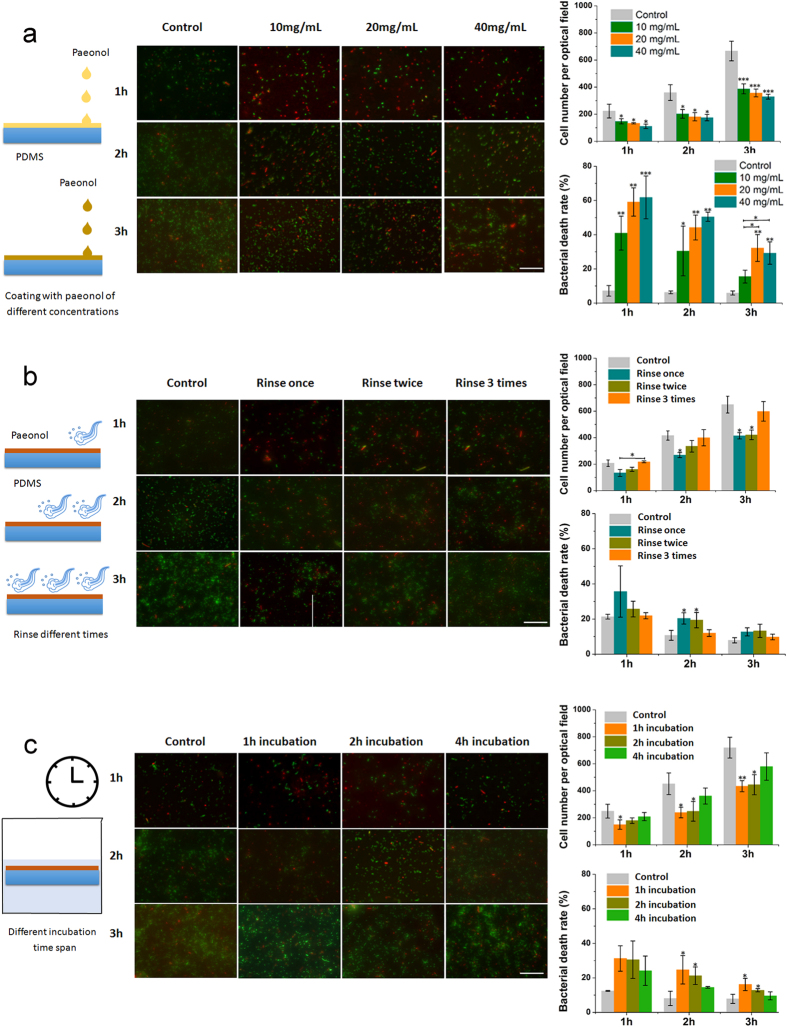
Stability of the paeonol-coated PDMS surface measured by its antibacterial effect against *E. coli* DH5α. Bacteria were observed 1, 2 and 3 h after culture. The uncoated PDMS surfaces were used as controls. (**a**) Antibacterial effect of the coated PDMS surfaces prepared with paeonol of different concentrations. (**b**) Antibacterial effect of the paeonol-coated PDMS surfaces against water rinsing. (**c**) Antibacterial effect of the paeonol-coated PDMS surfaces after incubation in water for different time spans. At least three experimental repetitions were carried out for each data point. The difference between sample groups and the control group was tested by Student’s *t*-test (*0.01 < p < 0.05, **0.001 < p < 0.01, ***p < 0.001). The length of scale bar is 100 μm.

Stability of the coating is crucial for the downstream application of PDMS. We measured the coating stability by quantifying the antibacterial effects. PDMS surfaces coated with paeonol solutions at concentrations ranging from 10 mg/ml to 40 mg/ml effectively inhibited the growth of *E. coli* on them. As the coating concentration increased, the relative bacterial number decreased and the bacterial death rate increased (Fig. 4a). However, the difference between different samples is within ca. 10%. In other words, coating PDMS with paeonol solutions at concentrations ranging from 10 mg/ml to 40 mg/ml produces PDMS surfaces with similar antibacterial effects. This result, in accordance with the WCA results (Fig. 1d), shows that our method is reliable with a high tolerance for paeonol coating concentration.

The coated PDMS surfaces were produced by being rinsed with water to remove excess paeonol. We further rinsed the coated PDMS surfaces additional 1 to 3 times or incubated them in water for additional 1–4 hours to test the stability of the coating (Fig. 4b and c). Compared with the uncoated PDMS surface, the paeonol-coated PDMS surface had a lower bacterial number and a higher cell death rate even after being rinsed or incubated in water. The result indicates the surface retained its satisfactory antibacterial effect. We also noticed that the antibacterial effect decreased gradually with rinsing and incubation. Nevertheless, the difference in antibacterial effect between the paeonol-coated PDMS surface and the uncoated PDMS surface was still obvious.

What should be noted here is that in our study, the coated PDMS surfaces were placed in bacteria culture medium. The concentration of bacteria here is much higher than in real conditions where a self-sterilized PDMS surface is needed. The paeonol-coated PDMS surface should exhibit better antibacterial effect in real-life applications.

### Sterilization of other surfaces by contact

**Figure 5:**
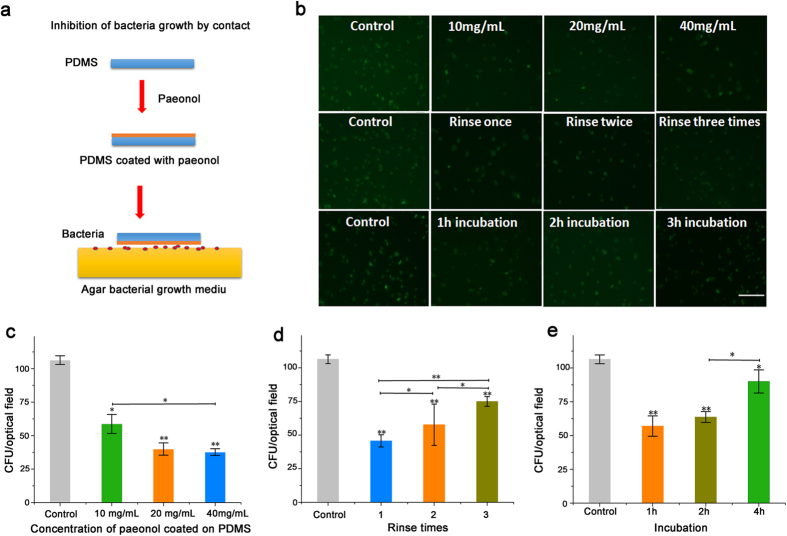
Antibacterial effect of the paeonol-coated PDMS surface to other surfaces by contact. (**a**) *E. coli* DH5α were seeded onto the agar bacteria culture plate. A disk of paeonol-coated PDMS was put onto the plate with the paeonol side towards the plate. The growth of bacteria beneath the paeonol-coated PDMS surface was measured by the number of bacteria colony forming units (CFU). (**b**) Fluorescent images of bacterial growth beneath the paeonol-coated PDMS surface under various experiment conditions. (**c**) Influence of paeonol-coating concentration on the growth of bacteria. (**d**) Antibacterial effect of the paeonol-coated PDMS surfaces on the contacting surface against rinsing. The paeonol-coated PDMS surfaces were rinsed with 1–3 times with water. (**e**) Antibacterial effect of the paeonol-coated PDMS surfaces on the contacting surface after incubation in water for 1 to 4 hours. At least three experimental repetitions were carried out for each data point. The difference between sample groups and the control group was tested by Student’s *t*-test (*0.01 < p < 0.05, **0.001 < p < 0.01). The length of scale bar is 100 μm.

It would be beneficial if the PDMS surface could also sterilize other surfaces by contact in biomedical devices. We showed that the paeonol-coated PDMS surface can not only self-sterilize but also sterilize other surfaces by contact. We seeded *E. coli* bacteria on an agar plate. Then the paeonol-coated PDMS disks were put onto the bacterial culture plate with the paeonol side towards the plate. We observed the colony forming unit (CFU) of the bacteria growing on the bacterial culture plate after 4 h. The CFU is a unit used to estimate the number of viable bacteria. In contrast with microscopic examination which counts all bacteria, living or dead, the appearance of a colony (counted as a CFU) in a cell culture requires significant cell growth. Compared to the bare PDMS surface, the paeonol-coated PDMS surface effectively decreased the number of CFU on the agar bacterial culture plate (Fig. 5). With the concentration of paeonol-coating solution increasing, the number of CFU kept decreasing continuously. Rinsing the paeonol-coated PDMS surface with water decreased its sterilization effect; however, an appreciable decrease in the number of CFU could still be observed. A similar result was found in the antibacterial test after incubation in water for 1–4 hours. Incubating the paeonol-coated PDMS surface in water for 4 h decreased its sterilizing effect on the contact surface; however an appreciable inhibition of bacterial growth was still observed.

### The paeonol-coated PDMS surface promotes normal mammalian cell growth but kills cancer cells

**Figure 6:**
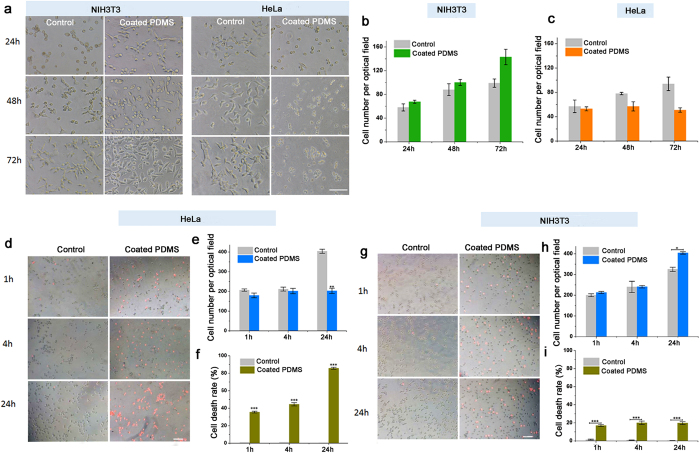
Growth of normal mammalian cells (NIH3T3) and cancer cells (HeLa) on the paeonol-coated and the uncoated PDMS surfaces. (**a**) Microscopic image of cell growth on the paeonol-coated and uncoated PDMS surfaces after cell culture for 72 h. The paeonol-coated PDMS surfaces promote NIH3T3 cell growth but inhibit HeLa cell growth. (**b**) Cell numbers of NIH3T3 cells growing on the paeonol-coated and the uncoated PDMS surfaces. NIH3T3 cells grow much better on the paeonol-coated PDMS surfaces. (**c**) Cell numbers of HeLa cells growing on the paeonol-coated and the uncoated PDMS surfaces. No increase of cell number is observed on the paeonol-coated PDMS surfaces. (**d**) Microscopic images showing the anticancer effect of the paeonol-coated PDMS surfaces after cell culture for 24 h. (**e**,**f**) are cell number and cell death rate of HeLa cells growing on the paeonol-coated PDMS surface, respectively. The uncoated PDMS surfaces were used as controls. (**g**) Microscopic images showing non-cancer NIH3T3 cell growth on the paeonol-coated PDMS surfaces after 24 h. (**h**,**i**) are cell number and cell death rate of NIH3T3 cells growing on the paeonol-coated PDMS surface, respectively. The uncoated PDMS surfaces were used as controls. The dead cells were stained using PI to be red. At least three experimental repetitions were carried out for each data point. The difference between sample groups and the control group was tested by Student’s *t*-test (*0.01 < p < 0.05, **0.001 < p < 0.01, ***p < 0.001). The length of scale bar is 100 μm.

Promoting mammalian cell growth on the PDMS surface is crucial for its applications as biomedical devices. We showed that the paeonol-coated PDMS surface promoted normal mammalian cell (NIH3T3) growth (Fig. 6a and b). The NIH3T3 cells grew much better on the paeonol-coated PDMS surface compared to the bare PDMS surface after 72 h cell culture (Fig. 6b). The number of NIH3T3 cells on the paeonol-coated PDMS surface was ca. 40% more than that on the bare PDMS surface. The morphology of NIH3T3 on the paeonol-coated PDMS surface was healthier than on the uncoated PDMS surface. The PDMS surface is reported to be unsuitable for mammalian cell growth due to its low surface energy and hydrophobicity, which inhibits cell adhesion^3^. Modification of PDMS surfaces with paeonol changes the surface wettability (as shown in Fig. 1d) and thus improves the cell growth on them.

In contrast, HeLa cancer cells could not grow on the paeonol-coated PDMS surface; although they grew poorly on the bare PDMS surface after cell culture for 72 h (Fig. 6a and c). Judging from the cell morphology, the HeLa cells were already dead after 72 h. In order to study the anticancer effect of the paeonol-coated PDMS surface over a shorter cell culture time, we specifically studied the growth of HeLa cells on the paeonol-coated PDMS surface after 24 h (Fig. 6d–f). NIH3T3 cells were used as a comparison (Fig. 6g–i). The number of HeLa cells did not increase and the cell death rate was close to 100% after 24 h cell culture. As a comparison, the death rate for HeLa cells growing on the uncoated PDMS surface was close to 0%. The result shows that the paeonol coating on the PDMS has a good anticancer effect. Meanwhile, the NIH3T3 cells grew rapidly and their cell number doubled after 24 h cell culture. The cell death rate for NIH3T3 cells on the paeonol-coated PDMS surface was ca. 5 times lower than for HeLa cells, which indicates the paeonol-coated PDMS surface did less harm to the non-cancer NIH3T3 cells. Although the paeonol-coated PDMS surface caused a slightly higher toxicity to non-cancer mammalian cells than the uncoated PDMS surface, at the same time it promoted their growth significantly. As a balance of the two effects, the growth of non-cancer NIH3T3 cells on the paeonol-coated PDMS surface was promoted as shown in Fig. 6a–c.

The anti-cancer effect of the paeonol-coated PDMS surface can probably be attributed to the regulatory effect of paeonol in the cell signal pathway. Paeonol can inhibit apoptosis in human cancer and has a synergistic effect by regulating the signalling pathways, including NF-κB, PI3K/PTEN/AKT and ERK/JNK/P38/MAPKs activations^11^. However, it is still unclear why non-cancer cells can survive the mechanism.

The Chinese herb *Paeonia moutan* has a long history of use as a traditional antibiotic medicine to cure inflammation^8^,^9^. In this study, we used four typical cells as models for Gram-positive bacteria, Gram-negative bacteria, cancer cells and normal cells. Nevertheless, there are hundreds of thousands of other bacteria, cancer cells and normal cells with different sensitivities to paeonol. It is too early to declare that paeonol can be used as a universal PDMS surface-coating material for antibacterial and anticancer applications, based simply on our results or the results using a few additional types of cells. A lot of work is still required to test whether the paeonol-coated PDMS surface is effective for new types of cells.

To sum up, we have successfully developed a method of modifying PDMS surface to provide it with multiple functions – antibacterial, anticancer and non-cancer mammalian cell growth promotion. Coating the PDMS surface with paeonol changes the surface wettability and element composition. Molecular dynamics simulation shows that the approach and adsorption of paeonol onto the PDMS surface is an energy favourable process. The paeonol-coated PDMS surface exhibits good antibacterial and anticancer activities against both Gram-negative and Gram-positive bacteria. The surface retains appreciable antibacterial effect after rinsing with water and incubation in water for hours. The antibacterial effect of the coated PDMS surface can occur on other surfaces by contact. Coating the PDMS surface with paeonol promotes non-cancer mammalian cell growth with low toxicity; however, it effectively inhibits cancer cell growth with a cell death rate close to 100%. Our study provides the first step for PDMS surface modification to achieve multiple functions, which potentially guides the application of the Chinese traditional herb in current scientific researches and the production of multifunctional PDMS devices.

## Materials and Methods

### Materials

The Chinese herb extract, paeonol, was obtained from Beijing Coupling Technology Co. Ltd. PDMS elastomer (Sylgard 184 silicone elastomer kit) was purchased from Dow Corning Corporation (Midland, MI). Propid-ium iodide (PI) was purchased from Sigma–Aldrich (China). Other reagents were purchased from Aladdin Reagent Co. Ltd. (China). All the reagents were of analytical grade. All aqueous solutions were prepared with double-distilled water. Genetically engineered *E. coli* DH5α expressing green fluorescent protein (GFP) and *P. aeruginosa* PAO1 strain were generously donated by Dr. Jun Feng, College of Life Science, Nankai University, Tianjin, China. HeLa cells (human cervical cancer cell line) and NIH3T3 cells (mouse fibroblast cell line) were generously donated by Miss Wenyan Han, College of Life Science, Nankai University, Tianjin, China.

### Preparation of paeonol**-**coated PDMS substrate

PDMS elastomer solutions, the base (part A) and the curing agent (part B), were mixed at a mass ratio of 10:1 in a glass dish (60 mm in diameter) and incubated at 65 °C for 1 day. The PDMS substrate was then washed with 75% ethanol and dried at room temperature. Paeonol-coating solution was dissolved in 75% ethanol solution. If not specifically mentioned, the concentration of paeonol-coating solution was 20 mg/ml. PDMS substrates were then immersed in the paeonol-coating solution and incubated for 3 h at room temperature. The paeonol-modified PDMS substrates were rinsed with double-distilled water and dried at room temperature. Both the paeonol-coated and native PDMS substrates were cut into round disks with a diameter of 7 mm. The thickness of the PDMS substrates was 1 mm.

### WCA and XPS Measurements

The WCAs were measured using a drop shape analysis instrument (Dataphysics, Inc., OCA20) under ambient conditions. The elemental composition on the PDMS surface was examined by an XPS (Thermofisher K-Alpha, USA) equipped with a monochro-matic Al Kα radiation source using a detection angle of 45°. Scans between 0 and 1350 eV were performed with an energy resolution of 0.9 eV.

### Molecular dynamics simulation

The initial structure of paeonol was built using GaussView 5.0^16^. The geometry optimization and frequency analyses on paeonol were performed using Gaussian 03 software^17^. Full geometry optimization was performed for each conformer at a level of B3LYP/6-31 G (d, p)^18^,^19^,^20^,^21^. Frequency analyses were warranted to achieve a minimum. The structure of the PDMS substrate was constructed using the VMD package^22^. The chemical formula for PDMS is Si(CH_3_)_3_O[Si(CH_3_)_2_O]_n_Si(CH_3_)_3_, where n is the number of repeat units. We used n = 20 in this study. A single PDMS chain was equilibrated in the gas phase for 10 ns. Then twelve PDMS conformers exacted from the trajectory in the last 2.4 ns (with intervals of 200 ps) were compressed into a slab of 55 × 55 × 11 Å^3^ according to experimental density by means of tclBC forces implemented in NAMD 2.9^23^. Finally, a full equilibration of this slab with a constant surface was performed using the periodic boundary conditions (PBCs) for another 10 ns^24^,^25^. A box containing 2197 water molecules together with 1 paeonol molecule was added onto the PDMS surface.

Atomistic MD simulations to simulate the interaction between paeonol and PDMS were performed using NAMD 2.9^23^. The force field parameters for the PDMS were taken from the available literature^26^, and the atom partial charges were taken from quantum chemistry calculations^27^. The parameters for paeonol were taken from CGenFF^28^,^29^. The TIP3 model was used for water molecules^30^. Long-range electrostatic interactions were treated by the Particle Mesh Ewald algorithm with a PME grid density of about 1.2 per grid point^31^. A 14 Å cutoff was used to calculate van der Waals interactions. Langevin pressure control was used to maintain a pressure of 1 bar. The piston period was 200 fs. The Langevin Piston Decay was 100 fs during the NPT simulation^32^,^33^. Langevin dynamics controlled the temperature at 300 K using a damping factor (5.0 ps^−1^) during the NPT simulation. The energy was minimized by 5,000 steps using the conjugate gradient method, SETTLE algorithm^34^, followed by NPT simulation for 10 ns. The time step was set to be 2 fs, and the coordinates were saved every 9.6 ps. Visualization and analysis of the MD trajectories was carried out with the VMD package.

### Antibacterial test in solution

The antibacterial activity of paeonol solution was evaluated by using *E. coli* DH5α and *P. aeruginosa* PAO1. Bacteria were cultured in LB medium at 37 °C for 12 h. The concentration of the bacterial suspension was 1 × 10^6^ cells/ml. The paeonol was added into LB medium to reach final concentrations of 0, 0.25, 0.50, 0.75 and 1 mg/ml, respectively. The relative density of bacteria was determined by the absorption (OD value) at a wavelength of 600 nm using UV-spectrophotometer (Shimadzu, UV-1800).

### Antibacterial test on the PDMS surface

Bacteria were harvested in the mid-exponential phase, centrifuged at 2,700 rpm for 10 min to remove the supernatant, and then re-suspended in a fresh medium^35^. The concentration of the bacteria suspension was 1 × 10^6^ cells/ml. The suspension (120 μL) was added to the paeonol-coated PDMS substrate and incubated for 1 h, 2 h and 3 h respectively at 37 °C. After incubation, the bacterial culture medium was removed. The *E. coli* DH5α (or *P. aeruginosa*) on the paeonol-coated and uncoated PDMS surfaces were stained with the red fluorescent nucleic acid dye propidium iodide (PI) (1.5 mM in H_2_O) for 5 min and then the surfaces were examined under a fluorescent microscope (TE 2000-U Nikon, Japan) to calculate the cell death rate.

### Mammalian cell culture on the paeonol-coated PDMS surface

The anticancer assay was studied using HeLa cells. HeLa cells were cultured in Dulbecco’s modified Eagle’s medium (DMEM), supplemented with 10% fetal bovine serum (FBS). The cells were cultured at 37 °C with 5% CO_2_. The paeonol-coated and the uncoated PDMS surfaces were placed on the bottom of 48-well plates. The initial cell seeding density was 3 × 10^4^ cells/well.

NIH3T3 cells were used as non-cancer mammalian cells. NIH3T3 cells were cultured in DMEM supplemented with 10% FBS. The cells were cultured at 37 °C with 5% CO_2_. The paeonol-coated and the uncoated PDMS surfaces were placed on the bottom of 48-well plates. The initial cell seeding density was 3 × 10^4^ cells/well.

## Additional Information

**How to cite this article**: Jiao, J. *et al*. Antibacterial and anticancer PDMS surface for mammalian cell growth using the Chinese herb extract paeonol(4-methoxy-2-hydroxyacetophenone). *Sci. Rep.* **6**, 38973; doi: 10.1038/srep38973 (2016).

**Publisher's note:** Springer Nature remains neutral with regard to jurisdictional claims in published maps and institutional affiliations.

## Supplementary Material

Supplementary Information
