# Supplementary material for: Antibacterial and anticancer PDMS surface for mammalian cell growth using the Chinese herb extract paeonol(4-methoxy-2-hydroxyacetophenone)
Source: Sci Rep. 2016 Dec 12;6:38973. doi: 10.1038/srep38973 (PMC5150582; doi:10.1038/srep38973)
Supplement: Supplementary Information [file srep38973-s1.pdf]

Supporting information

Antibacterial and anticancer PDMS surface for mammalian cell growth using the Chinese herb extract paeonol(4-methoxy-2-hydroxyacetophenone)

Jiajia Jiao[a,†], Lili Sun[b,†], Zaiyu Guo[c,†], Sen Hou[b], Robert Holyst[b,], Yun Lu[c,*], Xizeng Feng[a,*]*

[a] *State Key Laboratory of Medicinal Chemical Biology, The Key Laboratory of Bioactive Materials, Ministry of Education, College of Life Science, Nankai University, Tianjin, 300071, China.*

[b] *Institute of Physical Chemistry Polish Academy of Sciences, Kasprzaka 44/52, 01-224 Warsaw, Poland.*

[c] *TEDA Hospital, No. 65 Third Avenue, Economic-Technological Development Area, Tianjin 300457, China.*

†*These authors contributed equally to this work.*

**Corresponding authors*: rholyst@ichf.edu.pl (R.Holyst); tedaluyun@163.com (Y.Lu); xzfeng@nankai.edu.cn (X.Feng)

Table S1. Relative element compositions of the uncoated and the paeonol-coated PDMS surfaces

	O1s (%)	C1s (%)	Si2p (%)
PDMS	27.82	46.98	25.20
Coated-PDMS	29.41	45.73	24.85

Fourier transform infrared spectroscopy (FTIR).

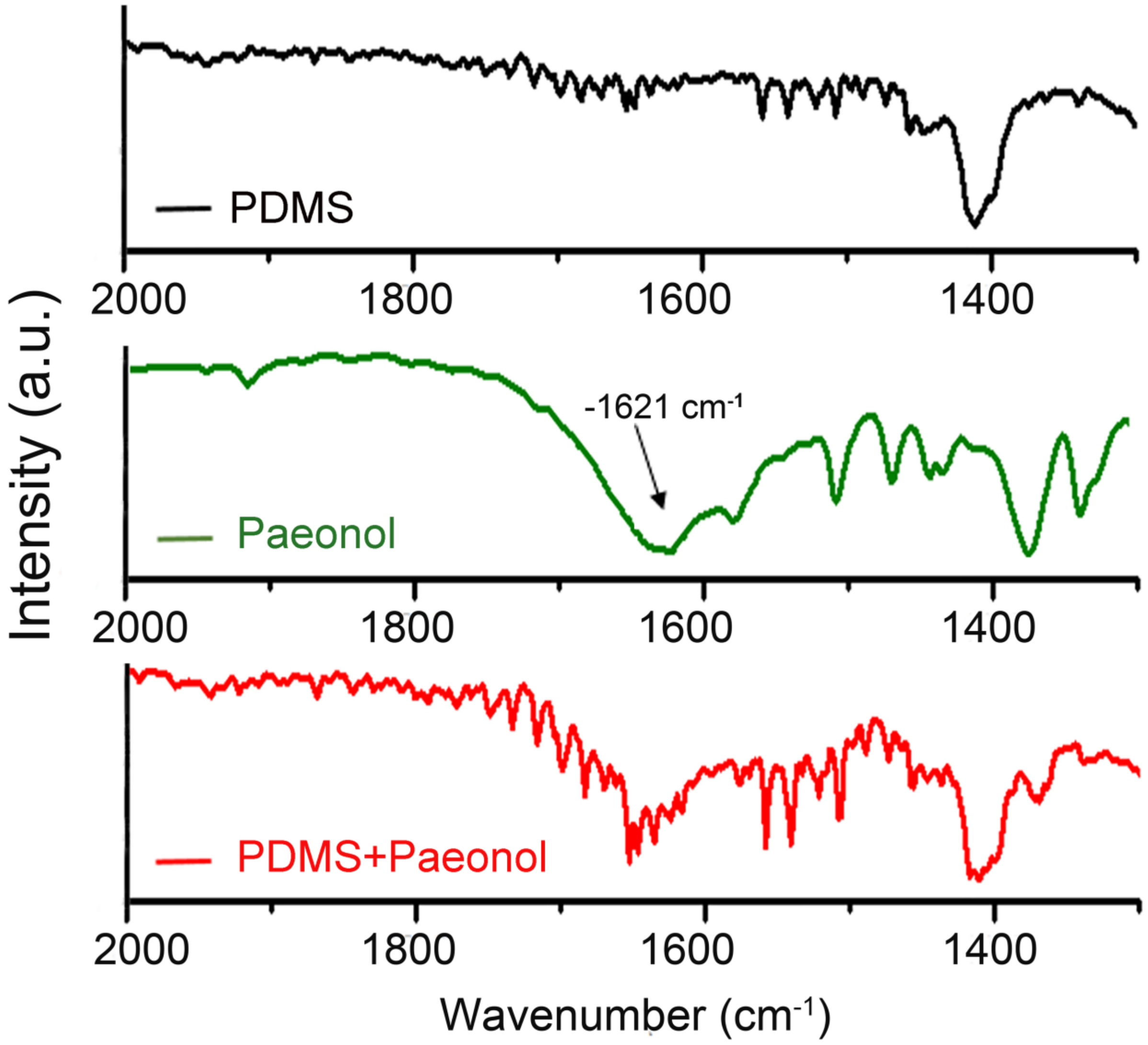

Figure S1. The FTIR spectra of the PDMS, the solid paeonol, and the paeonol-coated PDMS, indicating adsorption of paeonol onto the PDMS surface.

The FTIR spectra of the PDMS, the solid paeonol, and the paeonol-coated PDMS were collected on a Nicolet 550-II FT-IR spectrophotometer (Nicolet, USA) at a resolution of 0.09 cm^{-1}. As shown in Fig. S1, the FTIR spectrum of solid paeonol has a unique signal peak at -1621 cm^{-1} due to the stretching of the aromatic C=C and carbonyl C=O. (Hydrogen bonds were formed between the hydroxyl and carbonyl groups, so that the carbonyl C=O stretching signal peak appears at -1621 cm^{-1}.) These chemical groups do not exist in PDMS, so the -1621 cm^{-1} peak does not exist in the FTIR spectrum of the uncoated PDMS surface. Such

a signal is, however, present on the paeonol-coated PDMS, indicating the adsorption of paeonol onto the PDMS surface.
